# Extraocular Haptic Redocking and Vitrector‐Assisted Posterior Haptic Manipulation as an Intraoperative Rescue for Intraocular Lens Decentration During the Yamane Technique

**DOI:** 10.1155/crop/9039544

**Published:** 2026-10-19

**Authors:** Mitsuru Otsubo, Shun Konno, Yoshitaka Takizawa, Ami Konno, Harumasa Yokota, Taiji Nagaoka

**Affiliations:** ^1^ Department of Ophthalmology Asahikawa Medical University Asahikawa Hokkaido Japan asahikawa-med.ac.jp; ^2^ Department of Ophthalmology Sapporo Medical University Sapporo Hokkaido Japan sapmed.ac.jp

**Keywords:** haptic redocking, intraocular lens decentration, intrascleral intraocular lens fixation, pars plana vitrectomy, posterior approach, Yamane technique

## Abstract

**Purpose:**

The aim of this study is to report extraocular unilateral haptic redocking as an intraoperative rescue for intraocular lens (IOL) decentration during the Yamane technique.

**Methods:**

A 51‐year‐old woman underwent pars plana vitrectomy, explantation of a dislocated posterior chamber IOL, and flanged intrascleral IOL fixation using the Yamane technique. After externalization of both haptics, IOL decentration was observed. One externalized haptic was redocked into the lumen of a 30‐gauge needle outside the eye, and the contralateral haptic manipulation was converted to a posterior approach under a wide‐angle viewing system. The contralateral haptic was manipulated with the assistance of a vitreous cutter and subsequently inserted into another 30‐gauge needle within the vitreous cavity, allowing repositioning of the decentered IOL. At 3 months postoperatively, uncorrected distance visual acuity improved to 20/15, and the IOL remained stable without clinically significant decentration.

**Conclusion:**

Extra ocular haptic redocking and vitrector‐assisted posterior haptic manipulation may be useful as a rescue technique for intraoperative IOL decentration during the Yamane technique.

## 1. Introduction

Scleral fixation of an intraocular lens (IOL) is an effective surgical option in cases where capsular support is inadequate during or after cataract surgery [1]. The flanged intrascleral IOL fixation technique using a 30‐gauge needle, as described by Yamane et al., has gained widespread acceptance as a sutureless method [2, 3].

Although the Yamane technique is effective, IOL tilt and decentration are recognized complications [4]. Intraoperative IOL decentration may occur after haptic externalization, requiring appropriate repositioning. Hung et al. previously described reinsertion of an externalized haptic into the needle lumen as a troubleshooting technique for flanged IOL fixation [5]. In this report, we describe a case of intraoperative IOL decentration during the Yamane technique that was successfully managed by extraocular redocking of one externalized haptic into a 30‐gauge needle, followed by posterior manipulation of the contralateral haptic with the assistance of a vitreous cutter.

## 2. Case Report

A 51‐year‐old woman was referred to our hospital with decreased vision in the right eye. She had undergone uneventful bilateral phacoemulsification with implantation of an extended‐depth‐of‐focus IOL (TECNIS Symfony, Johnson & Johnson Vision, Santa Ana, California, United States, +20.5 D in the right eye and +21.0 D in the left eye) 6 years earlier at a local clinic. Nd:YAG laser posterior capsulotomy had been performed in the right eye 3 years prior to presentation.

Two days before referral, she experienced a sudden decrease in vision in the right eye. A dislocated IOL was identified at a local clinic, and she was referred to our institution for surgical management.

At presentation, the uncorrected distance visual acuity (UDVA) was 20/1000 in the right eye and 20/16 in the left eye. The best‐corrected visual acuity (BCVA) in the right eye was 20/16 with a +10.00 D correction. The intraocular pressure was 15 mmHg in the right eye and 13 mmHg in the left eye. Slit‐lamp examination demonstrated posterior chamber IOL dislocation in the right eye (Figure 1). The corneal endothelial cell density was 2772 cells/mm^2^. Pars plana vitrectomy, explantation of the dislocated IOL, and flanged intrascleral IOL fixation using the Yamane technique were performed.

**Figure 1:**
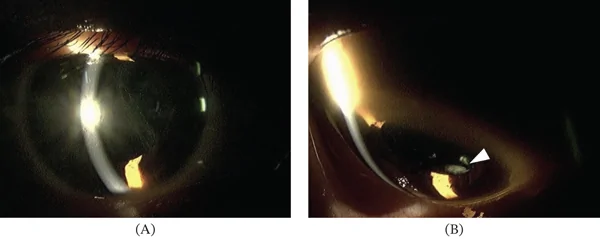
Preoperative photographs of the anterior segment. (A) The intraocular lens (IOL) is not visible in the primary position. (B) On downgaze, part of the dislocated IOL–capsular bag complex is observed (white arrow).

### 2.1. Surgical Technique

The surgery was performed under sub‐Tenon anesthesia. The surgical video of this case is provided (see Video, Supporting Digital Content 1, surgical video summary (available here)). A 25‐gauge vitrectomy system (Constellation Vision System; Alcon, Geneva, Switzerland) was used.

Three 25‐gauge trocars were inserted through the pars plana, and an additional 27‐gauge trocar was placed for chandelier illumination. Vitrectomy was performed under a wide‐angle viewing system (Resight; Carl Zeiss Meditec, Germany). The dislocated IOL was identified in the vitreous cavity and removed using an IOL cutter (Figure 2A).

**Figure 2:**
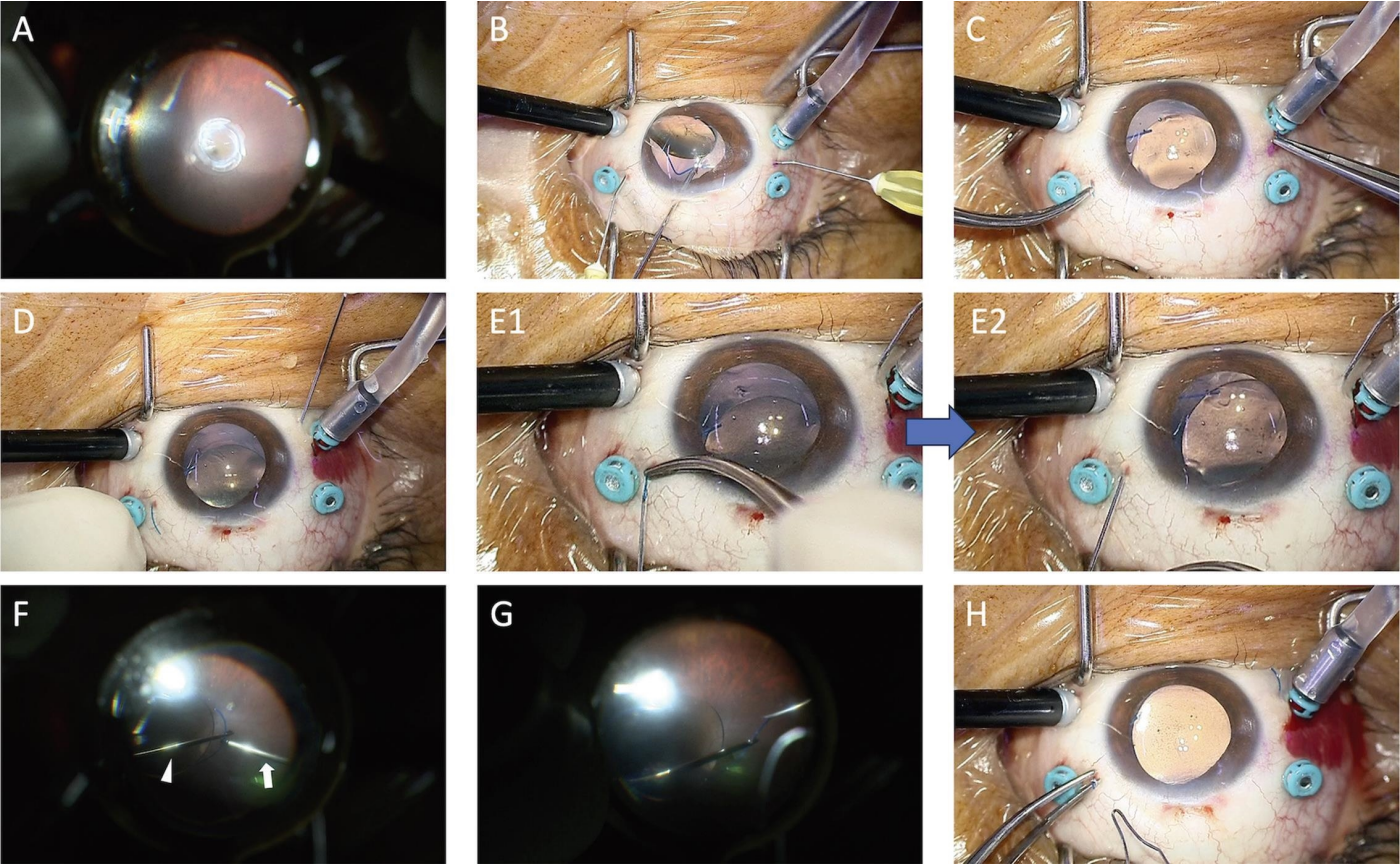
Intraoperative images: (A) A completely dislocated intraocular lens (IOL) is observed. (B) After IOL removal, a three‐piece IOL is implanted using the Yamane technique. (C) After haptic externalization, IOL decentration is observed. (D) A 30‐gauge needle is inserted at the 4‐o′clock position. (E1,E2) The externalized haptic at the 2‐o′clock position is redocked into the lumen of the 30‐gauge needle outside the eye, and the needle is reinserted through the sclera. (F) Under the wide‐angle viewing system, the haptic is grasped with vitreous forceps (white triangle) with assistance from the vitreous cutter (white arrow). (G) Under the wide‐angle viewing system, the haptic is inserted into the lumen of the 30‐gauge needle within the vitreous cavity. (H) After haptic externalization, improvement of the IOL decentration is confirmed.

Two 30‐gauge needles were inserted transconjunctivally through the sclera 2 mm posterior to the limbus. A 3‐piece IOL (NX‐70S; Santen Pharmaceutical Co. Ltd., Osaka, Japan) was inserted into the anterior chamber. The leading haptic was inserted into the lumen of the left‐sided 30‐gauge needle. The trailing haptic was grasped with intraocular forceps and inserted into the right‐sided needle (Figure 2B). After externalization of both haptics, intraoperative IOL decentration was observed (Figure 2C). Review of the surgical video revealed that the eye had remained in a downward position during insertion of the 30‐gauge needles, likely resulting in displacement of the intended scleral fixation sites.

Another 30‐gauge needle was then inserted at the 4‐o′clock position on the contralateral side, 2 mm posterior to the limbus (Figure 2D). Subsequently, the externalized left haptic was redocked into the lumen of the 30‐gauge needle outside the eye (Figure 2E1,E2)). The right haptic was returned into the vitreous cavity and manipulated under a wide‐angle viewing system using a posterior approach. The haptic was then inserted into the lumen of the 30‐gauge needle within the vitreous cavity. During manipulation of the haptic in the vitreous cavity, the optic portion of the IOL interfered with forceps access. Therefore, the vitreous cutter was used to assist haptic grasping and stabilization (Figure 2F,G). After externalization of the haptic, improvement of the IOL decentration was confirmed (Figure 2H). Approximately 2 mm of each haptic was trimmed, and a flange was created using low‐temperature cautery. The flanged haptics were then buried within the scleral tunnels. A peripheral iridotomy was then created, and the surgery was completed.

### 2.2. Postoperative Course

At 3 months postoperatively, the UDVA improved to 20/15 in the right eye. The IOL remained stable without clinically significant decentration (Figure 3). The corneal endothelial cell density was 2864 cells/mm^2^. No postoperative complications were observed.

**Figure 3:**
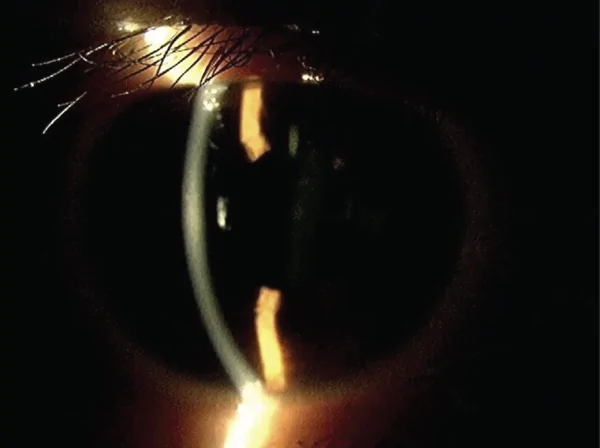
Postoperative anterior segment photograph at 3 months. The IOL remained stable without clinically significant decentration.

## 3. Discussion

In the present case, intraoperative IOL decentration was observed after externalization of both haptics during the Yamane technique. The decentration was successfully managed by extraocular redocking of one externalized haptic into a 30‐gauge needle, followed by posterior manipulation of the contralateral haptic within the vitreous cavity.

Review of the surgical video revealed that the eye had remained in a downward position during insertion of the 30‐gauge needles, likely resulting in displacement of the intended scleral fixation sites. Accurate positioning of the scleral fixation sites is important for achieving appropriate IOL centration in the Yamane technique [6]. In this case, determining the needle insertion sites while the eye was not in the primary position may have contributed to the subsequent IOL decentration. Therefore, confirming the primary position of the eye before determining the needle insertion sites may help prevent this complication.

When IOL decentration is recognized after externalization of both haptics, repositioning can be technically challenging because both haptics are outside the eye, and the IOL is no longer supported by the needles. Hung et al. previously described a double‐needle haptic repositioning technique in which an externalized haptic is reinserted into a 30‐gauge needle and the needle‐haptic complex is returned into the eye through the original scleral tract [5]. In the present case, we similarly redocked one externalized haptic into a 30‐gauge needle outside the eye. This maneuver provided temporary control of one side of the IOL while the contralateral haptic was repositioned through a posterior approach.

Posterior approaches for haptic manipulation have previously been described by Erakgun and Iannetti et al. Erakgun described the “intravitreal needle technique,” using a 27‐gauge needle for fixation of posteriorly dislocated three‐piece IOLs, whereas Iannetti et al. reported a posterior approach using a 30‐gauge needle for repositioning of a dislocated IOL [7, 8]. In the present case, the posterior approach was not used as the primary fixation technique but was adopted intraoperatively after IOL decentration occurred. Following extraocular redocking of one haptic, the posterior approach allowed manipulation of the contralateral haptic and repositioning of the IOL without explantation of the newly inserted IOL. Chandelier illumination was essential during the posterior approach because both grasping of the contralateral haptic and its insertion into the 30‐gauge needle required bimanual manipulation. The IOL optic partially obstructed grasping of the contralateral haptic with a single vitreous forceps. Although haptic transfer using two vitreous forceps, as in the handshake technique, could also have been considered [9], we instead gently pushed the haptic toward the IOL optic with the port of the vitreous cutter, facilitating its subsequent grasping with vitreous forceps (Figure 2F). This approach also required fewer forceps grasps of the haptic and may reduce the risk of haptic deformation.

When IOL decentration is recognized after haptic externalization, repositioning through a corneal incision may increase the risk of contact with the corneal endothelial or iris. In the present case, conversion to a posterior approach allowed manipulation of the contralateral haptic within the vitreous cavity under a wide‐angle viewing system. Favorable postoperative visual acuity was achieved without an apparent decrease in corneal endothelial cell density.

This report has several limitations. First, this was a single‐case report with only short‐term follow‐up. Second, the posterior manipulation used in this rescue procedure requires chandelier illumination because bimanual manipulation is necessary. In addition, the posterior approach requires pars plana vitrectomy and trocar placement for manipulation within the vitreous cavity. Therefore, this technique may be particularly suitable for cases of dislocated IOLs requiring simultaneous vitreoretinal surgery.

## 4. Conclusion

Extraocular redocking of an externalized haptic may be useful as a rescue technique for intraoperative IOL decentration during the Yamane technique.

## Funding

No funding was received for this manuscript.

## Ethics Statement

Ethical approval was not required for this single case report. Written informed consent was obtained from the patient for publication of the clinical information, images, and surgical video.

## Consent

Written informed consent was obtained from the patient for publication of this case report and accompanying images and surgical video.

## Conflicts of Interest

Mitsuru Otsubo, Shun Konno, Yoshitaka Takizawa, and Ami Konno declare no conflicts of interest. Harumasa Yokota has received financial support from Alcon and research funding from Santen Pharmaceutical, Novartis, Bayer Yakuhin, AMO Japan, and Senju Pharmaceutical. Taiji Nagaoka has received financial support from Daicel, Ribomic, LTT Bio‐Pharma, and Kowa.

## Supporting information


**Supporting Information** Additional supporting information can be found online in the Supporting Information section. File S1: Digital Content 1.mp4.

## Data Availability

The data that support the findings of this study are available from the corresponding author upon reasonable request.
